# Factors influencing the bariatric surgery treatment of bariatric surgery candidates in underdeveloped areas of China

**DOI:** 10.1186/s12893-024-02373-8

**Published:** 2024-03-05

**Authors:** Xiangxin Kong, Yuan Zhang, Ruoer Li, Lei Yang, Yin Xian, Ming He, Ke Song, Aimei Jia, Qin Sun, Yixing Ren

**Affiliations:** 1 Department of Gastroenterology, Affiliated Hospital of North Sichuan Medical College, Nanchong, 637000 China; 2 North Sichuan Medical College, Nanchong, 637000 China

**Keywords:** Factors, Overweight, Obesity, China, Bariatric surgery, Economic factors

## Abstract

**Background:**

From year to year, the proportion of people living with overweight and obesity in China rises, along with the prevalence of diseases linked to obesity. Although bariatric surgery is gaining popularity, there are still several issues with its promotion compared to Western nations. Since less developed places in China are more widespread due to disparities in the development of different regions, there has been little exploration of the factors that might be related to acceptance of bariatric surgery in these regions.

**Methods:**

Patients who visited the Department of Gastrointestinal Surgery at the North Sichuan Medical College Affiliated Hospital from 2018 to 2022 and had obesity or other relevant metabolic problems were surveyed using a questionnaire. The relationship between demographic factors, socioeconomic status, and acceptance of bariatric surgery was analyzed.

**Results:**

Of 334 patients, 171 had bariatric surgery. BMI, education level, marriage history, medical insurance, family support, and a history of type 2 diabetes were all linked to having bariatric surgery, according to a univariate analysis. In a multivariate analysis, BMI (*P* = 0.02), education (*P* = 0.02), family support (*P*<0.001), medical insurance coverage (*P*<0.001), and history of type 2 diabetes (*P* = 0.004) were all positively associated with a willingness to have bariatric surgery. Among 163 non-bariatric patients with obesity, 15.3% were not opposed to surgery but preferred trying medication first, 54.6% leaned towards medical therapy, and 30% were hesitant. Additionally, a majority of patients (48.55%) often lacked adequate knowledge about weight reduction therapy. Age, height, gender, smoking, drinking, family history of type 2 diabetes, education, and marital status did not significantly differ (*P* > 0.05).

**Conclusions:**

Many patients are concerned about the safety of surgical treatment and the possibility of regaining weight. Due to the relatively high cost of bariatric surgery, they tend to choose medical treatment. To enhance the acceptance of bariatric surgery in underdeveloped regions of China, it is crucial to focus on disseminating knowledge about bariatric surgery, offer pertinent health education to the community, and foster support from patients’ families. The government should pay more attention to obesity and provide support in the form of medical insurance.

**Supplementary Information:**

The online version contains supplementary material available at 10.1186/s12893-024-02373-8.

## Introduction

Obesity is a growing global public health issue and a national epidemic China [1, 2]. In 2022, the percentage of people living with overweight and obesity adults in China is projected to reach 50.7% [3]. As early as 2014, China had already become the country with the highest number of individuals living with obesity [4]. Research indicates that bariatric surgery offers a durable and efficient solution for weight loss [5]. Moreover, compared to simple lifestyle adjustments and medical management, the effectiveness of bariatric surgery as a treatment for people with type 2 diabetes (T2D) and obesity is more effective [5, 6]. In the past, development of bariatric surgery in China has lagged behind that of European and American nations because few people lived with overweight or obesity and there has been limited societal acceptance of bariatric surgery.

As the notion that bariatric surgery can ameliorate obesity and its related ailments gains traction, an increasing number of patients with obesity are opting for this form of treatment. Nevertheless, due to the nascent stage of bariatric surgery in China, there is limited interest among Chinese patients with obesity, resulting in low adoption rates [7]. Additionally, in Chinese culture, there is a common misconception that having excess body fat signifies good health and prosperity [8]. This misconception also hinders the widespread adoption of bariatric surgery in China. With the rapid advancement of my country’s economy and society, economic activities have exhibited increasing disparities. Economic endeavors have progressively concentrated in specific regions, leading to the swift economic progress of eastern coastal cities. Consequently, a discernible disparity persists in economic prowess between China’s central and western regions compared to the eastern coastal areas. This segment of the country is considered underdeveloped [9–12]. Due to variations in regional development, underprivileged areas may have limited access to information and less robust economies. This circumstance could potentially hinder their willingness to undergo bariatric surgery as a treatment for obesity.

The acceptability of this novel treatment approach among candidates for bariatric surgery in China has been investigated but only in developed areas so any differences from underdeveloped areas remain unknown [13]. There remains a substantial population of patients with obesity in underdeveloped areas of China who have not undergone bariatric surgery. Our research is dedicated to formulating more precise promotion plans, hoping to know what measures can be taken to encourage people to accept this form of treatment, and to provide a theoretical basis for the promotion of bariatric surgery.

## Methods

### Patients

From 2018 to 2022, patients with obesity-related metabolic disorders (including obesity and related conditions) who met the criteria for Bariatric surgery and resided in underdeveloped areas of China (e.g., Gansu, Guizhou, Yunnan, Qinghai, Sichuan, Tibet, Shaanxi, Ningxia Hui, Xinjiang Uygur, Inner Mongolia) were evaluated at the First Department of Gastrointestinal Surgery, Affiliated Hospital of North Sichuan Medical College. They received a follow-up questionnaire addressing factors like race, economic status, family support, education, etc. They have resided in underdeveloped regions for over a year.

The study team members contacted the subjects to explain the purpose of the study, answer any questions, and review the informed consent forms. If the person agreed to participate, members of the same study team who obtained consent immediately began the questionnaire. During the process of organizing and counting the questionnaires, neither the questionnaire collector nor the questionnaire statistician knew whether the patient had undergone bariatric surgery. The manner of data collection and analysis was consistent during the period of the study. All subjects answered the questionnaire (Appendix I). The responses of subjects who underwent bariatric surgery were compared with those who did not undergo bariatric surgery (the reference group in the multivariate analysis. All subjects who completed the questionnaire met the minimum criteria for bariatric surgery outlined in the 2022 IFSO-APC Consensus Statement [14].

The questionnaire was initially formed based on reference to relevant literature [13, 15–17]. Prior to the formal survey, we conducted a pilot study with 50 patients with obesity and incorporated their feedback and comments into the questionnaire. The questionnaire consisted of four sections. The initial section covered participants’ general demographic information, including gender, age, height, weight, source of surgical information, marital status, medical insurance status, and employment status. The second section evaluated participants’ knowledge about obesity, its associated metabolic disorders, and family medical history. The third section explored attitudes towards surgery and weight loss medications. For the detailed questionnaire, refer to the appendix I.

### Statistical analysis

All clinical data were analyzed by SPSS (version 13.0) statistical software (IBM SPSS Inc., Armonk, New York, United States: IBM Corp). Chi-square tests were used to analyze whether there were differences in demographic characteristics, social status, and past medical history between patients who underwent surgery and those who did not. On the basis of a univariate analysis, variables were chosen for the multivariate logistic regression model, which provided odds ratio (OR) and 95%CI for association with whether bariatric surgery was performed. *P* < 0.05 was considered statistically significant.

## Results

We administered a total of 500 questionnaires; 200 respondents had undergone surgery. We received 342 completed questionnaires. After removing some surveys with incomplete responses, 334 were deemed valid. This resulted in an overall response rate of 66.8% (85.5% in the surgery group and 54.3% in the control group). This experiment included 334 subjects from underdeveloped areas in China, including 123 males (65 underwent surgery, 52.85%) and 211 females (106 underwent surgery, 50.24%). A statistical comparison of the subjects who did and did not undergo surgery is shown in Table 1.

**Table 1:** Comparison of factors in underdeveloped regions

	Surgical group (*n* = 171)	Non-surgical group (*n* = 163)	*p*
**Gender**	*N*(%)	*N*(%)	**0.645**
Male	65(38.01)	58(35.58)	
Female	106(61.99)	105(64.42)	
**BMI category**			**0.002**
< 28	9(5.26)	4(2.45)	
28-34.9	109(63.74)	56(34.36)	
35-39.9	37(21.64)	72(44.17)	
40-49.9	14(8.19)	30(18.40)	
≥ 50	2(1.17)	1(0.61)	
**Age (years)**			**0.078**
≤ 17	1(0.58)	4(2.45)	
18–40	130(76.02)	108(66.26)	
≥ 40	40(23.39)	51(31.29)	
**Employment status**			**0.969**
Unemployed	78(32.75)	74(45.40)	
Employed	93(67.25)	89(54.60)	
**Education**			**0.002**
Illiterate	0(0.00)	3(1.79)	
Primary school	10(5.85)	16(10.71)	
Middle school	49(28.65)	29(17.86)	
High school	48(28.07)	31(8.93)	
University/above	64(37.43)	84(60.71)	
**Marriage**			**<0.001**
Single	25(14.62)	92(56.44)	
Married	144(84.21)	52(31.9)	
Divorced/widowed	2(1.17)	19(11.66)	
**Medical insurance coverage**			**<0.001**
No	45(26.32)	107(65.64)	
Yes	126(73.68)	56(34.36)	
**Family support**			**<0.001**
Yes	161(94.15)	107(65.64)	
No	10(5.85)	56(34.36)	
**Smoking**			**0.327**
Yes	32(18.71)	42(25.77)	
No	139(81.29)	121(74.23)	
**Alcohol consumption**			**0.742**
Yes	14(8.19)	15(9.20)	
No	157(91.81)	148(90.80)	
**Family history of Type 2 diabetes**			**0.145**
Yes	72(42.11)	56(30.36)	
No	99(57.89)	107(69.64)	
**History of type 2 diabetes**			**0.004**
Yes	91(53.22)	61(32.14)	
No	80(46.78)	102(67.86)	
**History of hypertension**			**0.529**
Yes	33(19.30)	36(22.09)	
No	138(80.70)	127(77.91)	

The analysis revealed that BMI (*P* = 0.02), education level (*P* = 0.001), marital history (*P* = 0.001), possession of medical insurance (*P* < 0.001), family support (*P* < 0.001), and history of T2DM (*P* = 0.004) significantly influenced a patient’s decision to undergo bariatric surgery (Table 1). Gender, age, employment status, smoking and drinking history, hypertension and a family history of type 2 diabetes mellitus (T2DM) were not statistically significant (*P* > 0.05).

Multivariate analysis showed that BMI (*P* = 0.002), education (*P* = 0.002), medical insurance coverage (*P* < 0.001), family support (*P* < 0.001), T2DM history (*P* = 0.004), and marriage (*P* < 0.001) were positively correlated with undergoing bariatric surgery. Age was not statistically significant (*P* > 0.05). Table 2.

**Table 2:** Multivariate logistic analysis of factors associated with choice of bariatric surgical treatment among patients in underdeveloped areas of China

Independent variables	OR	95%CI	*p*
**BMI, kg/m2**	2.098	1.556–2.827	<0.001
**Education**	1.114	0.904–1.373	0.309
**Medical insurance coverage**	5.350	3.346–8.555	<0.001
**Family support**	8.426	4.118–17.240	<0.001
**History of type 2 diabetes**	1.902	1.229–2.944	0.004
**Marriage**	2.745	1.823–4.133	<0.001

Additionally, we investigated the treatment preferences of patients in the non-surgical group. More than half of them expressed a willingness to try medication for treating obesity. Interestingly, 30.06% of patients were not inclined towards either surgical or medical treatment. Moreover, 15.34% of patients showed an interest in surgical intervention for obesity, yet they were more inclined towards medical treatment (Table 3).

**Table 3:** Treatment preferences of bese subjects in non-surgical group (*n* = 163)

Treatment preferences	*n*	%
interested in surgery but preferred to try meds first	25	15.34
More likely to receive medical treatment	89	54.60
Do not receive medical treatment or surgical treatment.	49	30.06

Questions that addressed hesitancy revealed that a substantial portion (48.47%) were largely unfamiliar with bariatric surgery. Among the rest, the predominant concern was about the safety of the surgery (25.77%). Additionally, 15.33% opted against bariatric surgery for obesity due to cost considerations, while another 10.43% expressed apprehension about the postoperative prognosis (Table 4).

**Table 4:** Reasons for reluctance of patients in the non-surgical group to undergo surgery (*n* = 163)

Reasons	*n*	%
Price	25	15.33
Safety	42	25.77
Surgical prognosis	17	10.43
Unfamiliarity	79	48.47

## Discussion

We believe that various factors affect the acceptance of weight-loss surgery by individuals in underdeveloped area of China, including limited access to information, low income, insufficient support from social insurance, and the availability of weight-loss drugs. Through multivariate logistic regression analysis, we discovered that education level does not directly influence patients’ willingness to undergo bariatric surgery. In a study on Chinese nurses’ perceptions and attitudes towards obesity and bariatric surgery, it was observed that nurses with a master’s degree or higher exhibit a greater understanding of obesity complications, and the incidence of obesity is lower compared to the national average [15]. Factors like BMI and medical insurance collectively influence patients’ decisions regarding bariatric surgery. Variables like BMI and medical insurance play a comprehensive role in influencing a patient’s decision regarding bariatric surgery, to further explore the reasons for these factors, we conducted a detailed discussion as follows:

### BMI

Our findings show that BMI is associated with bariatric surgery acceptance. However, compared with other articles on the acceptance of bariatric surgery in the Chinese population in developed areas, we found that the understanding of obesity and bariatric surgery in underdeveloped regions of China was insufficient [18]. As indicated by the survey in Table 4, among patients with obesity who have not undergone bariatric surgery, 48.47% were unfamiliar with bariatric surgery. BMI correlates with people’s physical health. Studies have shown [19] that BMI is positively correlated with metabolic syndrome, Compared with people with a healthy BMI (18.5-28 kg/m^2^), those living with a BMI (> 28 kg/m^2^) had a 67%, 58%, and 74% higher risk of developing metabolic syndrome, diabetes, and hypertension, respectively. Therefore, BMI is one of the main factors influencing patients’ choice of bariatric surgery. Thus, in the process of promoting bariatric surgery, it is crucial to also disseminate information about the correlation between BMI and metabolic conditions associated with obesity. This will enhance public awareness regarding weight management.

### Medical insurance coverage

The results of our study showed patients with medical insurance were more likely to accept surgery (Table 2). To adapt to different peoples, the China insurance system differs for urban and rural areas, covering almost all of China. However, obesity has not yet been fully accepted by patients as a disease in China, as indicated by the fact that bariatric surgery in some provinces of China is not eligible for reimbursement, which undoubtedly is a considerable burden to patients [13]. So, assistance with the financial aspects of the surgery is critical to the decision. On the other hand, in some parts of the West, it is even more pronounced, with access to bariatric surgery being highest among those with private insurance and lowest among those with public health insurance, a phenomenon that seems to be related to the Insurance coverage for illness [20]. Therefore, the ability of patients to afford surgery with proper health insurance also has a significant impact on their access to surgery. According to reports, over the past 30 years, more than half of the global increase in body mass index (BMI) has been due to the increase in BMI in rural areas of middle- and low-income countries, and obesity is an important factor in causing a range of metabolic diseases [4]. Therefore, we should address this disease by providing governmental support for patients with obesity, reducing the cost of some medical supplies, and alleviating the burden on patients.

### Family support

Family support plays a key role in patients undergoing surgery [21–23] These studies demonstrate that familial support during the surgical process not only boosts the patient’s confidence pre-operatively, but also aids in post-operative recovery. A patient’s decision to have surgery is under pressure in many ways, but having parental support gives patients more courage. Our results showed that most patients who underwent surgery had familial support. In the few patients who did not receive familial support, the parents said bariatric surgery was too risky. As reported in the literature, current data suggest that perioperative mortality in bariatric surgery ranges from 0.03–0.2% [24]. Therefore, doctors should vigorously emphasize the safety of this surgery throughout the country, especially in underdeveloped areas to reduce patient and parental anxiety. Furthermore, we observed that being married increases the likelihood of undergoing bariatric surgery, possibly because these patients may place greater importance on their appearance in the eyes of their spouses. Bariatric surgery can indeed enhance a patient’s appearance. Additionally, their spouses provided support and encouragement. Therefore, in the process of educating patients with obesity about weight loss, it is crucial to also engage with their family members. This way, family members can play a role in motivating and supporting patients in their weight loss journey.

### Medical treatment

We observed that just 25 (15.3%) of 163 individuals with obesity who did not have bariatric surgery were interested in surgery but preferred to try meds first compared to 54.6% who were more inclined to get medical therapy and 30% who were hesitant to undergo surgery (Table 3). There is no denying that there has been some progress in drug-based treatments for metabolic syndrome in recent years. GLP-1 receptor agonists have been found in intensive studies of obesity to improve glycemic control and lead to weight loss, so they have received increasing attention in the treatment of diabetes-obesity [25]. Among the patients in our survey who were unwilling to undergo surgical treatment, 48.6% did not understand the treatment for weight loss, and 26% of the patients were more concerned about the safety of surgical treatment (Table 4). Apart from stimulating glucose-dependent insulin secretion, intravenous GLP-1 receptor agonists suppress glucagon release, slows gastric emptying, induces an early and acute decrease in appetite and food intake. Moreover, it lowers blood pressure, mitigates inflammation, reduces coagulation, and facilitates natriuresis and diuresis [26, 27]. Additionally, gliflozin represents a novel category of oral antidiabetic medications, acting as inhibitors of sodium-glucose co-transporters (SGLTs). They work by decreasing glucose absorption in the proximal tubule and intestinal epithelium [28]. Previous studies have demonstrated that SGLT-2 inhibitors and GLP-1 receptor agonists also lead to reduced mortality rates, as well as a decrease in specific cardiovascular and renal events [28, 29]. GLP-1 receptor agonists has been proven to be effective in weight loss, but for some diabetic patients, the degree of weight loss and anti-diabetic effects are not as good as bariatric surgery, in mainland China, GLP-1 receptor agonists has not yet received approval for treating obesity. Therefore, the effectiveness and safety of this type of drug for Chinese patients with metabolic syndrome require further verification, and the requirement for patient compliance is higher [30, 31]. In the short term, bariatric surgery remains a safe and effective method to treat metabolic syndrome in East Asia. Therefore, for patients who have not undergone bariatric surgery but are amenable to drug treatment, we should conduct long-term follow-ups on the treatment’s effectiveness for metabolic syndrome. At the appropriate juncture, we can recommend bariatric surgery to enhance patient acceptance of obesity treatment.

### Obesity-related symptoms and comorbidities

Our investigation found that a family history of T2DM was not statistically significant in the consideration of bariatric surgery, but a previous history of T2DM was positively correlated with an interest in bariatric surgery (*P* = 0.004). Therefore, health conditions may motivate a patient to choose bariatric surgery. This result is consistent with previous studies that did not differentiate between developed and less developed areas of China [13]. We believe that these patients with obesity, after developing obesity-related diseases and receiving treatment in the hospital, were introduced to the concept that bariatric surgery and convinced that it can address both obesity and its related metabolic conditions. This could explain why this group of patients opted for bariatric surgery. In addition, our results show that a personal history of hypertension was not statistically significant in choosing to undergo bariatric surgery. Bariatric surgery, in fact, yields superior results in improving blood pressure for patients with obesity and with hypertension compared to conventional hypertension treatment [32].We believe that for less developed areas, more patients may feel that their medical history has no bearing on the operation, and the interaction between diseases is not clear. People living with obesity who may have type 2 diabetes or hypertension in less developed areas are not well understood about the relationship between these complications and living with obesity, and therefore are reluctant to receive such treatments. Therefore, we suggest that more health education activities should be carried out to spread knowledge about obesity and obesity-related complications, and to let patients know which diseases are related to obesity.

### Others: smoking, alcohol consumption, education, and marriage

In addition to the above factors, we found that marital history and education level were positively associated with undergoing surgery(*P* = 0.07, *P* = 0.002, respectively), while smoking and drinking were not statistically significant. In marital status, patients with a family (84.2%) were more likely to undergo surgery, suggesting that support and encouragement from their partner helped them feel more confident. Today, 6 million people die each year from tobacco-related diseases [25]. Even if it is not statistically significant in patients undergoing surgery, smoking should not be ignored. Spreading the idea of tobacco control to patients, whether they have surgery or not, can improve their health when older and prevent other complications. The same is true of alcohol consumption.

## Conclusions

Many factors affect patient decisions in underdeveloped areas on whether to undergo surgery; the primary factor is concern about the risks of surgery. As a result, doctors must first enhance their professional skills, then properly advise patients about the safety of surgery and do a thorough preoperative evaluation. Surgeons should converse with patients to learn their reasons for declining surgery, then take steps to address those factors based on patient concerns. Medical professionals can better help patients find ways to overcome these obstacles. Second, the expansion of medication treatment has influenced patients’ decisions to undergo bariatric surgery. Candidates for bariatric surgery in less developed areas typically favor medication treatment because of aspects including surgical safety and cost. Compared with more developed areas, for patients with obesity in less developed areas, the most important thing is to inform them of the dangers of living with obesity and to promote obesity-related drugs and bariatric surgery. It is equally important for the popularity of bariatric surgery to increase their acceptance of bariatric surgery. The government should also increase support for patients with obesity to some extent and increase the proportion of reimbursement.

### Electronic supplementary material

Below is the link to the electronic supplementary material.

Supplementary Material 1

## Data Availability

All data generated or analyzed during this study in included in this published article.
