# Supplementary material for: Factors influencing the bariatric surgery treatment of bariatric surgery candidates in underdeveloped areas of China
Source: BMC Surg. 2024 Mar 5;24:82. doi: 10.1186/s12893-024-02373-8 (PMC10913241; doi:10.1186/s12893-024-02373-8)
Supplement: Supplementary file 1 — Supplementary Material 1 [file 12893_2024_2373_MOESM1_ESM.docx]

**Questionnaire**

(Fill in the blanks and type in the appropriate brackets “√”)

**一、basic information:**  File number:

1、Name: 2、Identity number:

3、Gender: Male ( ) Female ( ) 4、Date of birth (Year / Month / Day):

5、Weight:（ ） Height:（ ）

6、 Phone (home): 7、Telephone ( Cell phone):

8、Family address:

9、Access to surgical information: Internet ( ) newspaper ( ) Mobile APP ( ) doctor（ ）

Family/ friends ( ) Other, please specify:_______________

10、 Does your family support your surgery?

Support（ ） Not support（ ） I didn't tell them（ ）

11、Education: none ( ) Primary school ( ) Junior high school ( ) High school ( ) College degree or above ( )

12、 Marital Status: Single（ ） Married（ ） divorced and widowed（ ）

1. Do you have health insurance to pay for weight loss and metabolic surgery?

Yes( ) No( )

14、Profession: Yes（ ） No（ ）

**二、Parents' social status and past history**

1. Medical history: none ( ) Yes, please specify:

2. Smoking History: No ( ) Yes( ) number of cigarettes ( ) Such as: (/day)

1. Drinking history: No ( ) Yes( ) number of cigarettes ( ) Such as: (/day)

4. Family history:

| Disease Father Mother grandparents brothers and sisters |
| --- |
| Coronary heart disease |
| Type 2 diabetes |
| Hypertension |
| Obesity |
| Other please specify |

5. Medical history:

History of type 2 diabetes: yes（ ） No（ ）

History of hypertension: yes（ ） No（ ）

1. **Have you undergone metabolic surgery? Yes（ ） No（ ）（Supplement: If you choose No, please answer the following questions）**

Which treatment would you prefer to receive non-surgical （ ）

A、more willing to take medication B、interested in surgery but preferred to try meds first C、neither want

**Choose A or C, please answer the following questions**

1. Reasons for reluctance to undergo surgery

The price is more cost-effective（ ） higher security（ ） better prognosis（ ） Don't know about obesity treatment（ ）
